# Impact of bottle size on in‐home consumption of wine: a randomized controlled cross‐over trial

**DOI:** 10.1111/add.15042

**Published:** 2020-04-08

**Authors:** Saphsa Codling, Eleni Mantzari, Olivia Sexton, Georgia Fuller, Rachel Pechey, Gareth J. Hollands, Mark Pilling, Theresa M. Marteau

**Affiliations:** ^1^ Behaviour and Health Research Unit University of Cambridge UK

**Keywords:** Alcohol, bottle size, consumption, cross‐over, portion size, randomized trial, RCT, wine

## Abstract

**Aim:**

To assess the impact of purchasing wine in 50 cl bottles compared with 75 cl bottles on the amount of wine consumed at home.

**Design:**

Cross‐over randomized controlled trial with a ‘usual behaviour’ period of a maximum of 3 weeks between conditions.

**Setting:**

Households in the United Kingdom.

**Participants:**

One hundred and eighty‐six households that consumed between two and eight 75 cl bottles of wine each week.

**Intervention:**

Households were randomized to the order in which they purchased wine in two bottle sizes. During two 14‐day intervention periods, households purchased a pre‐set volume of wine—based on their baseline self‐reported weekly consumption—in either 75 cl bottles or 50 cl bottles. On days 7 and 14 of each study period, participating households sent photographs of each purchased wine bottle.

**Measurements:**

The primary outcome was the volume of study wine in millilitres (ml) consumed during each study period estimated through returned photographs. The secondary outcome was the rate of consumption measured by the mean number of days taken to drink 1.5 litres from each bottle size.

**Findings:**

One hundred and sixty‐six of 186 enrolled households satisfactorily completed the study. After accounting for pre‐specified covariates, 191.1 ml [95% confidence interval (CI) = 42.03–339.2] or 4.5% (95% CI = 1.0–7.9%) more wine was consumed per 14‐day period from 75‐cl bottles than from 50‐cl bottles. Consumption was 5.8% faster (95% CI = –10.9 to –0.4%) from 75 cl bottles than from 50 cl bottles.

**Conclusions:**

Consuming wine at home from 50 cl bottles, compared with 75 cl bottles, may reduce both amount consumed and rate of consumption.

## Introduction

Alcohol consumption is a major contributor to premature death and disease globally [1], with increases in consumption since 1990 predicted to continue [2]. Approximately 3 million deaths per year world‐wide and 290 000 per year throughout Europe can be attributed to the consumption of alcohol [3], much of which occurs in homes [4, 5]. Reducing alcohol consumption among populations would decrease the incidence of non‐communicable diseases, including some cancers, cardiovascular diseases and type 2 diabetes [6].

The most effective interventions for reducing alcohol consumption are those that target contextual factors of affordability, availability and advertising [7, 8, 9, 10, 11]. Another potential target for intervention is the size of containers—e.g. bottles and glasses—in which alcohol is packaged, sold and served. This follows from the results of a Cochrane systematic review, which found that larger portions and packages increase the consumption of food and non‐alcoholic drinks [12]. No studies were found that related to alcoholic drinks. Recent field studies show that larger glasses increase wine consumption in restaurants [13]. To date, no studies have assessed the impact of wine bottle size upon consumption.

Wine is the most commonly drunk alcoholic beverage in Europe, including Britain, with most wine being bought in stores to consume at home rather than in pubs, bars and restaurants [14, 15, 16]. A bottle containing 75 cl is now widely accepted as the standard for wine [17]. More recently, smaller bottles have become more widely available in countries, such as the United Kingdom [18, 19, 20]. The impact of smaller bottles on consumption is unknown. Smaller bottles have the potential to decrease consumption by increasing the effort required to open and consume more than one bottle [12], or as a result of a tendency for people to consume a specific number of bottles in any one drinking occasion, regardless of bottle size [21]. Smaller bottles could also increase consumption by reducing barriers to consumption that are present for larger sizes [22], including any inhibitions over opening larger packages, perhaps increasing the frequency of drinking episodes. Furthermore, as 75 cl wine bottles have become the norm, the amount of wine held in smaller bottles may be perceived as too small. Indeed, studies suggest that visual exposure to larger portion sizes may adjust perceptions of what constitutes a ‘normal’‐sized portion [23]. If smaller bottles are perceived as too small, this could inadvertently lead to overconsumption of wine, as additional bottles are opened and consumed during a drinking episode [24, 25].

The results of a feasibility and acceptability study, comparing how drinkers respond to 75 cl and 37.5 cl bottles, highlighted the possibility that the amount held in the smaller bottles might be considered too small, thus potentially increasing consumption [26]. This counterproductive consequence could be minimized with 50 cl wine bottles, which have become more readily available in UK supermarkets [18], since this feasibility study was conducted. Constituting two‐thirds, as opposed to half the size of a standard wine bottle, 50 cl bottles may be more effective than 37.5 cl bottles at decreasing wine consumption on any one drinking occasion, if not overall.

The current randomized controlled cross‐over trial addresses the absence of evidence regarding the potential effectiveness of smaller bottles to reduce wine consumption. The aims of the study were to compare purchasing of a fixed volume of wine in 50 cl bottles to 75 cl bottles to assess the impact on [1] the amount of wine consumed at home; and [2] the rate of wine consumption at home.

## Methods

The study protocol was prospectively registered with the Open Science Framework (OSF) on 20 September 2018 (https://osf.io/gvndq), and retrospectively registered with ISRCTN on 8 November 2018: ISRCTN16597253 (https://doi.org/10.1186/ISRCTN16597253). The statistical analysis plan was specified prior to data analysis and uploaded to OSF (https://osf.io/rjzmf/) on 19 July 2019. Data will be available from the Open Science Framework with other study materials (together with the study protocol and statistical analysis plan, already uploaded) upon publication.

The study was approved by the University of Cambridge Psychology Research Ethics Committee (reference no: PRE.2018.064). Written informed consent to participate in this study was obtained by representative members of all participating households.

### Study design

The study was a randomized controlled cross‐over trial with a ‘usual behaviour’ period between conditions (75 cl bottles versus 50 cl bottles). The design was informed by a feasibility study [26]. A cross‐over design was chosen due to the expected variability between households in terms of size, number and characteristics of wine drinkers. Being able to control for these differences made a cross‐over design the most efficient use of resources to address the study aims.

### Participants

Data were collected from 186 households in the United Kingdom between October 2018 and June 2019. Eligible households were those in which adult members had the following characteristics: (i) self‐reported that they collectively drank a minimum of 2 × 75 cl bottles of wine a week; 1 (ii) lived within a 3‐mile radius of a store that stocked the study wine in both 50 cl and 75 cl bottles; (iii) were in possession of a device, such as a smartphone, from which to take and send photographs of wine consumed; (iv) did not take medications for which there was a recommendation against alcohol consumption; (v) did not have a serious mental illness (e.g. paranoia, schizophrenia or other psychotic disorder; bipolar disorder or schizoaffective disorder, assessed by self‐report by asking participants to tick any condition they might have had) or history of extreme alcohol abuse [assessed by self‐report (‘Do you have any history of alcohol abuse?’ yes/no)] or of becoming ill enough to require hospitalization after alcohol consumption; and (vi) agreed to consume the study wine for the duration of the study. 2

Potentially eligible households were recruited by a research agency (Roots Research; https://rootsresearch.co.uk/) from their panel of participants, as well as advertisements on social media and approaching individuals on the street.

### Sample size

The study was powered to detect a meaningful effect size of a 250 ml difference in household consumption between bottle sizes. This difference is based on each wine drinker per household drinking one fewer small glass of wine (125 ml) per 14‐day period, given an average of two wine drinkers per household (rounded‐up to the nearest whole person), as observed in a feasibility study [26]. To detect this difference using the variance of differences observed in the feasibility study [standard deviation (SD) = 1235.4 ml] with 80% power and an alpha of 5%, 154 households were required to complete the cross‐over study. To account for possible attrition, 186 households were recruited.

### Randomization

Households were randomized to the order in which they purchased wine in the two different bottle sizes. Randomization occurred in Qualtrics, an on‐line survey platform, during completion of a baseline online questionnaire. Blocked randomization was used to ensure an even split between households receiving either the 50 cl or the 75 cl bottles first.

### Intervention

The intervention comprised purchasing a fixed quantity of wine in one of two bottle sizes: (i) 75 cl and (ii) 50 cl. This is a size × product intervention within the TIPPME intervention typology (typology of interventions in proximal physical micro‐environments) [27].

Study wines were chosen based on their availability in the same brand and grape variety in both target sizes from a specified retailer (see Supporting information, Data S1 for study wine list). Occasionally, due to stocking problems, a small number of wines could only be matched on brand.

The quantity of wine purchased by each household was derived from the household baseline self‐reported volume of wine consumed at home per week. This weekly volume was multiplied by 3, to provide sufficient wine for 3 weeks’ supply. The volume was then rounded‐up to the nearest multiple of 1.5 litres, to ensure that the same quantity could be purchased in both bottle sizes.

Each intervention period lasted 2 weeks (14 days). There was an intervening ‘usual behaviour’ period of up to 3 weeks, with a longer duration permitted in some circumstances, to allow households to finish the wine ordered during the first intervention period. Households were able to start the second intervention period as soon as the wine from their first intervention period had been, or was close to being, finished.

### Procedure

Full details of the study procedures sent to participants are provided in Supporting information, Data S2. These include a study time‐line, outline and a video link explaining how to take days 7 and 14 photographs.

Representatives of potentially eligible households, i.e. individuals recruited from each household to liaise with the study team and provide data, were directed to a baseline questionnaire to assess their eligibility. All the study questionnaires were conducted on‐line, using the Qualtrics survey platform. In an attempt to mask the true aim of the study, participants were given a cover story that the study was exploring the impact of bottle size on the experience of wine drinking, including taste. All household representatives provided written consent before study enrolment.

Eligible households were then randomized to their first condition—i.e. to purchase wine in either (i) 75 cl or (ii) 50 cl bottles—and told the volume and quantity of bottles they should purchase. Representatives were asked to select the brand/varieties of wines their household would like to receive for the first intervention period before being redirected to the retailer's website for purchasing. Household representatives were then asked to e‐mail their order confirmation to the research team.

Upon receipt of order confirmations, the research team posted out study instructions along with self‐adhesive labels to attach to the study wine bottles (Supporting information, Data S2). The labels were designed to record the following: (i) the date each bottle was opened and finished; (ii) the number of household members and guests who drank from the bottle; and (iii) the estimated volume that guests drank. Households were also asked to record the volume of any non‐study wine consumed at home.

Household representatives sent photographs to the researchers of all (labelled) bottles on days 7 and 14 of both intervention periods. Each household representative received an e‐mail reminder on the day photographs were due, with a further reminder sent the following day if necessary. Photographs were checked upon receipt, with any queries followed‐up directly with households. Once photographs were approved, participants were e‐mailed a questionnaire to report out‐of‐home wine consumption and any mitigating factors they thought had affected in‐home consumption, such as sickness during the study period. Households that finished their wine before the end of the study period could purchase more study wine in volumes that were multiples of 1.5 litres. 3

At the end of the first 14‐day intervention period, households returned to their ‘usual behaviour’ to allow any remaining study wine to be finished. During this period, there were no constraints on bottle size or types of wine that households could drink.

Once household representatives confirmed that they had consumed all their wine or were close to having consumed all their wine, they were e‐mailed a second questionnaire directing them to the wine bottle size and quantity they were required to buy for their second 14‐day intervention period. Households were required to order the same volume, but not necessarily the same wine brands that they had ordered for the first intervention period.

At the end of the study, all household representatives were invited to complete an end‐of study questionnaire comprising five questions on what they thought the study was about and their attitudes towards the two bottle sizes. Household representatives were then fully debriefed on the true aim of the study.

Households received £240 in total for completing the study in full. There was no additional compensation for the wine purchases made during the study.

### Outcome measures

#### Primary outcome

Volume of study wine consumed (in millilitres) during each intervention period for each bottle size (75 versus 50 cl), which was estimated through returned photographs of all study wine bottles purchased. Full details on the procedures followed to determine consumption from photographs of partially empty bottles are provided in the Statistical Analysis Plan (https://osf.io/rjzmf/).

#### Secondary outcome

The mean number of days taken to consume each 1.5 litres of wine during each intervention was estimated from the study start‐ and finish‐dates reported on submitted photographs.

#### Covariates

The following nine covariates were used in the analyses:

1. *Non‐study wine consumption*: in‐home consumption of non‐study wine (in millilitres) by the household during each of the 14‐day intervention periods, assessed by self‐reports on bottle labels.
2. *Guest consumption*: guest consumption of study wine (in millilitres) during each of the 14‐day intervention periods, assessed by self‐reports on bottle labels.
3. *Out‐of‐home consumption*: out‐of‐home wine consumption (in millilitres) by household members during each intervention period, assessed by self‐reports on questionnaires on days 7 and day 14 of each intervention period.
4. *Number of wine drinkers in household*: number of wine drinkers in a household, as self‐reported in the baseline questionnaire.
5. ‘*Usual behaviour*’ period duration: duration of the ‘usual behaviour’ period (in days) per household.
6. *Baseline consumption*: volume (in millilitres) of wine consumed per week per household at baseline, self‐reported in the baseline questionnaire.
7. *Price per litre*: mean price (£) per litre of all ordered wine.
8. *Guessed study aim*: awareness of study aim, assessed in the end‐of‐study questionnaire through free text responses to a question asking participants what they thought the study was about.
9. *Mitigating factors*: i.e. any noteworthy events or circumstances external to the study, self‐reported in a free text field by participants as having increased or decreased their wine consumption during each intervention period. This was measured via questionnaires completed at days 7 and 14 of each intervention period. These were coded independently by two researchers, from −1 (decreasing consumption) to +1 (increasing consumption) for each 7‐day period in an intervention period and combined to gain a single total variable per 14‐day period, ranging from −2 to +2.

#### Other measures

Other measures comprised demographic characteristics of households (mean household age, number of adults, annual household income) and of each household's representative (age, gender, education, ethnicity), all self‐reported by the participating households' representatives.

### Statistical analysis

Demographic characteristics of households and household representatives completing the study were described [means (SDs); proportions (%)] and compared to those who enrolled in the study but did not complete it. The mean volume of wine ordered in the two bottle‐size conditions was calculated as a manipulation check, to ensure that volumes ordered with the two bottle sizes were consistent and any differences in consumption were not due to differences in available volumes.

To assess the impact of bottle size on the primary outcome, a mixed‐effects regression analysis was performed on the ‘as per‐protocol’ sample, fitting household as a random factor, controlling for all pre‐specified covariates. Standard cross‐over design covariates included in the model were variables for the order in which the two bottle sizes were purchased and time (i.e. first or second intervention periods). All regression model diagnostics were checked [i.e. residual, quantile–quantile (QQ) and influence plots] and were satisfactory. Analysis was per‐protocol due to the cross‐over design of the study.

Four sets of sensitivity analysis were conducted to assess the robustness of the impact of bottle size on the primary outcome by separately adding the following to the analysis: (i) all households that were randomized, i.e. intention‐to‐treat analysis (ITT); (ii) households that completed the study in the reverse order to their randomization; (iii) whether or not households had guessed the study aims; and (iv) self‐reported mitigating factors influencing consumption.

For the secondary outcome, a cumulative sum for the total volume of wine consumed was calculated per day. This was used to calculate the number of days taken to drink: (i) 1.5 litres, (and for households that drank more); (ii) from 1.5 to 3 litres; (iii) from 3 to 4.5 litres; and (iv) from 4.5 to 6 litres. These were averaged to calculate the mean number of days taken to drink 1.5 litres. Data were not normally distributed and log‐transformation was required to satisfy modelling assumptions.

To assess the impact of bottle size on the log‐transformed mean number of days to drink 1.5 litres, a mixed‐effects regression analysis was performed, fitting household as a random factor, with the same covariates as for the primary analysis. Coefficients were exponentiated to obtain the percentage change and 95% confidence intervals (CI) were calculated.

### Content analysis

Responses given on the end‐of‐study questionnaire were analysed with a focus on perceptions towards the 50 cl bottles and were coded in terms of their overall tone as either positive, negative or neutral/mixed. Main themes and subthemes across comments were identified and classified as either positive, negative or neutral/mixed.

### Patient and public involvement

The design and implementation of the study, including the plans for recruitment and measurement of the outcomes, were independent of patients and the public. Patients or members of the public were not invited to comment on the study design or contribute to the writing or editing of this document for readability or accuracy. The results of the research will be shared with the general public through internet news, popular science articles and social media.

## Results

**Figure 1:**
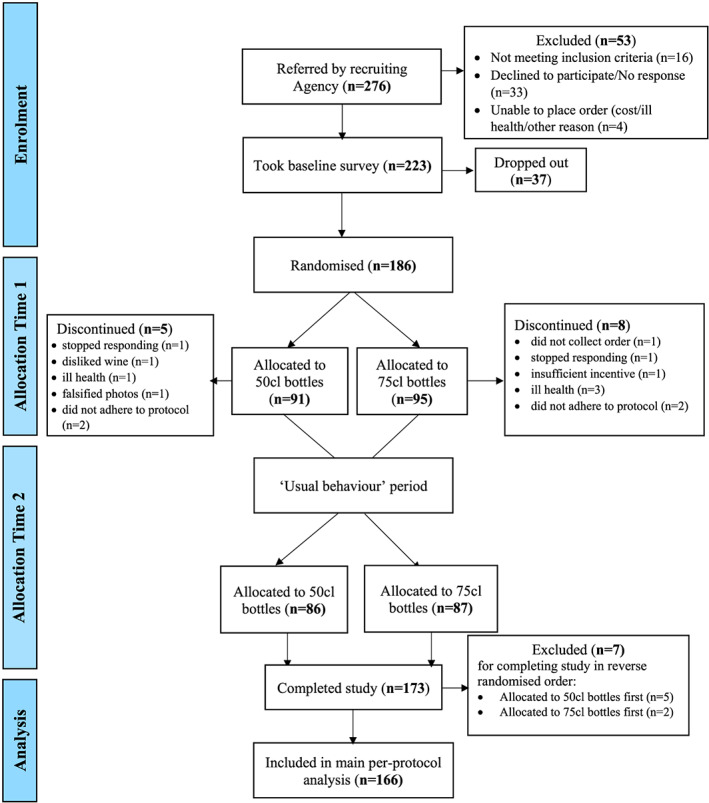
Flow of households throughout the study. [Colour figure can be viewed at wileyonlinelibrary.com]

The flow of households through the study is shown in Fig. 1. Two hundred and seventy‐six eligible households were identified by the research agency as being eligible for enrolment. All were assigned a study ID and 166 completed the study, as per protocol. Characteristics of households completing the study and household representatives—i.e. the individuals who consented to take part in the study and provided data on behalf of their households—are shown in Table 1. The households and their representatives were broadly comparable to consumers of alcohol in Britain, the majority of whom are white and of higher socio‐economic position, as indicated by income and occupation [28]. The drinking patterns of the sample were also comparable to those of English alcohol drinkers, who in 2017 reported that their average weekly alcohol consumption was 11.8 units [29]. 4

**Table 1:** Characteristics of (a) households (adults) and (b) household representatives completing the study per protocol (*n* = 166), according to intervention order.

	50 cl first (*n* = 84)	75 cl first (*n* = 82)	Overall (*n* = 166)		
(a) Households					
No of adults (mean, SD)	2.1 (0.9)	2.2 (0.9)	2.2 (0.9)
Age (adults in household) (mean, SD)	39.0 (10.3)	38.4 (10.2)	38.8 (10.1)
Sex (mean %, SD)			
Female	48% (2.8)	48% (2.6)	48% (2.7)
Male	48% (2.9)	45% (2.6)	46% (2.7)
Not reported	2%	7%	4%
No of wine drinkers (mean, SD)	1.9 (0.7)	1.9 (0.6)	1.9 (0.7)
No. of 75 cl bottles of wine consumed per week (mean, SD)	2.6 (0.9)	2.6 (0.7)	2.6 (0.8)
Annual household income (*n*, %)			
< £15 k	2 (3%)	2 (2.5%)	4 (2%)
£15–25 k	10 (12%)	6 (7%)	16 (10%)
£25–35	17 (20%)	7 (9%)	24 (14%)
£35–50 k	14 (17%)	17 (21%)	31 (19%)
£50–70 k	17 (20%)	27 (33%)	44 (27%)
> £70 k	22 (26%)	20 (25%)	42 (25%)
Prefer not to say	3 (2%)	2 (2.5%)	5 (3%)
(b) Household representatives					
Age (mean, SD)	39.4 (8.6)	38.5 (9.4)	38.9 (8.9)	
Sex (*n*, %)				
Female	59 (69%)	60 (74%)	119 (72%)	
Male	26 (31%)	21 (26%)	47 (28%)	
Highest level of education (*n*, %)				
Below A‐levels^a^	13 (15%)	18 (22%)	31 (19%)	
A levels or vocational training	21 (25%)	11 (14%)	32 (19%)	
Bachelor's degree and above	51 (60%)	52 (64%)	103 (62%)	
Ethnicity (*n*, %)				
White	77 (91%)	76 (94%)	153 (92%)	
Black	3 (3.5%)	1 (1%)	4 (2%)	
Asian	1 (1%)	3 (4%)	4 (2%)	
Mixed	4 (5%)	1 (1%)	5 (3%)	

a A‐levels are equivalent to a US high school diploma, a French Baccalauréat or a German Abitur. SD = standard deviation.

Compared to those who enrolled into the study and completed it as per protocol (*n* = 166), households that discontinued participation mid‐study (*n* = 13) drank significantly more bottles of wine per week (*t*_(12.769)_ = 2.526, *P* = 0.042) and included adults who were significantly younger (*t*_(15.333)_ = −3.510, *P* = 0.003). Household representatives were also younger (*t*_(14.931)_ = −2.767, *P* = 0.014), more likely to be male and more likely to have a lower level of education (Fisher's exact test, *P* = −0.01) There was no evidence that non‐completers had different levels of income to completers (Fisher's exact test, *P* = 0.73). The characteristics of the households and household representatives discontinuing participation are shown in Table 1 in the Supporting information, Data S4.

Descriptive information regarding the primary and secondary outcomes and covariates according to bottle size and intervention order are shown in Table 2. There was no significant difference in the mean volume of wine ordered per household in each bottle size [50 cl bottles: 6135.5 ml (SD = 2207.0); 75 cl bottles: 6122.0 ml (SD = 2197.6); paired*‐t*_(165)_ = 0.763, *P* = 0.463], evidence that households adhered to the instructions to order the same volume of wine during each study period.

**Table 2:** Study outcomes and covariates (mean, SD) during each 14‐day intervention period according to intervention order (*n* = 166).

	Bottle size phase					
	50 cl			75 cl		
	50 cl first	75 cl first	Overall	50 cl first	75 cl first	Overall
Primary outcome						
Volume of study wine consumed (ml)	4262.5 (2054.9)	4147.9 (2076.7)	4205.9 (2060.2)	4281.5 (2253.5)	4478.0 (1981.0)	4378.6 (2119.1)
Secondary outcome						
Mean number of days taken to consume 1.5 litres of study wine	5.44 (2.60)	4.96 (2.10)	5.21 (2.37)	4.67 (2.88)	4.60 (2.23)	4.63 (2.57)
Covariates						
(i) Non‐study wine consumption (ml)	212.5 (838.5)	143.3 (511.3)	178.3 (695.1)	81.5 (431.9)	291.2 (787.4)	185.1 (693.8)
(ii) Guest consumption (ml)	472.9 (656.4)	463.7 (918.1)	468.4 (794.1)	354.5 (570.0)	560.9 (910.3)	456.4 (762.1)
(iii) Out‐of‐home consumption (ml)	785.1 (1215.9)	615.1 (936.9)	701.1 (1087.1)	690.9 (1272.1)	553.8 (743.1)	622.8 (1042.4)
(iv) Number of wine drinkers	1.86 (0.75)	1.94 (0.63)	1.90 (0.71)	1.86 (0.75)	1.94 (0.63)	1.90 (0.72)
(v) ‘Usual behaviour’ period duration (days)	9.83 (6.41)	9.95 (6.76)	9.89 (6.56)	9.83 (6.41)	9.95 (6.76)	9.90 (6.57)
(vi) Baseline consumption (ml)	1950.9 (726.5)	1929.9 (543.6)	1940.5 (640.9)	1950.9 (726.5)	1929.88 (543.6)	1940.5 (640.9)
(viii) Price (£) per litre	8.75 (1.44)	8.50 (1.27)	8.62 (1.36)	8.19 (1.53)	7.9026 (1.49)	8.04 (1.52)
(ix) Mitigating factors	−0.19 (1.06)	−0.20 (1.20)	−0.19 (1.13)	−0.20 (1.00)	0.13 (1.01)	−0.04 (1.02)

SD = standard deviation.

### Primary outcome: volume of wine consumed

The volume of wine consumed with the 75 cl bottles was 172.7 ml higher per household per 14‐day period compared to the 50 cl bottles (Table 2; Supporting information, Plot 1, Data S6). This appears to be due primarily to a reduction in consumption in the second period by the group who purchased 75 cl bottles first, although the difference in effect between the two groups was not statistically significant. After adjusting for pre‐specified covariates, models showed that bottle size had a significant impact on volume consumed, with 191.1 ml (95% CI = 42.03–339.2) more wine consumed per 14‐day period from 75 cl bottles than from 50 cl bottles (Table 3; Supporting information, Plot 2, Data S6). Given the total volume of wine consumed across households per 14‐day period (4292 ml), this equates to a 4.5% (95% CI = 1.0 to 7.9%) increase in consumption with 75 cl bottles compared to 50 cl bottles.

**Table 3:** Mixed‐effect regression model estimates (95% CI) for volume (ml) of wine consumed per 14‐day period (*n* = 166).

				95% CI for estimate	
	Estimate (SE)	*t*‐value	*P*‐value	Lower	Upper
Intercept	277.8 (547.5)	0.51	0.612	−781.7	1337.8
Bottle size 75 cl (ref: 50 cl)	191.1 (76.5)^*^	2.49	0.013	42.03	339.2
Intervention period (ref: period 1)	−80.5 (74.1)	−1.08	0.278	−225.1	63.04
Intervention order (ref: 50 cl first)	32.8 (197.0)	0.17	0.878	−348.5	413.7
Baseline consumption (ml)	2.24 (0.16)^**^	13.8	< 0.001	1.93	2.56
Guest consumption (ml)	0.55 (0.09)^**^	5.75	< 0.001	0.36	0.74
Out‐of‐home consumption (ml)	−0.03 (0.06)	−0.56	0.578	−0.15	0.08
Non‐study wine consumption at home (ml)	0.004 (0.074)	0.05	0.956	−0.14	0.15
Price (£) per litre	−4.41 (40.0)	−0.11	0.912	−82.8	72.9
‘Usual behaviour’ period duration (days)	−56.7 (15.1)^**^	−3.77	< 0.001	−85.9	−27.6
Number of wine drinkers in household	−28.4 (149.2)	−0.19	0.849	−316.9	260.4

* Significant at the *P* < 0.05 level;

** significant at the *P* < 0.01 level. CI = confidence interval; SE = standard error.

Baseline consumption, guest consumption and the duration of the ‘usual behaviour’ period each had a significant impact on consumption volume. More wine was consumed per 14‐day intervention period when households self‐reported higher baseline levels of wine consumption or had guests who drank from their study wine, with less wine consumed for each extra day spent in the ‘usual behaviour’ period (Table 3).

### Secondary outcome: consumption rate

After adjusting for pre‐specified covariates, bottle size had a significant impact on consumption rate, with consumption being 5.8% faster (−5.8%: 95% CI = –10.9 to –0.4%, with smaller values showing faster rates) from 75 cl bottles than from 50 cl bottles (Table 4).

**Table 4:** Mixed‐effect regression model, logged estimates and estimates expressed in terms of percentage change (95% CI) for rate of wine consumption^a^ (*n* = 166).

	Estimate (SE)	*t*‐value	*P*‐value	95% CI for estimate		
				Lower	Upper	Percentage change (95% CIs)
Intercept	2.15 (0.16)	13.7	< 0.001	1.85	2.46	
Bottle size 75 cl (ref: 50 cl)	−0.06 (0.03)^*^	−2.1	0.040	−0.12	−0.00	−5.8% (−10.9 to −0.4%)
Intervention period (ref: period 1)	0.01 (0.03)	0.4	0.691	−0.04	0.07	1.1% (−4.3 to 6.8%)
Intervention order (ref: 50 cl first)	−0.02 (0.05)	−0.5	0.646	−0.11	0.07	−2.2% (−10.7 to 7.2%)
Baseline consumption (litres)	−0.41 (0.04)^**^	−10.2	< 0.001	−0.48	−0.33	−33.4% (−38.3 to −28.1%)
Guest consumption (litres)	−0.14 (0.03)^**^	−4.9	< 0.001	−0.19	−0.08	−13.0% (−17.7 to −8.0%)
Out‐of‐home consumption (litres)	0.01 (0.02)	0.5	0.622	−0.03	0.05	0.9% (−2.7 to 4.7%)
Non‐study wine consumption at home (litres)	0.004 (0.026)	−0.01	0.889	−0.05	0.05	−0.4% (−5.3 to 4.8%)
Price (£) per litre	0.02 (0.01)	1.7	0.089	−0.00	0.05	2.3% (−0.3 to 4.9%)
‘Usual behaviour’ duration (days)	0.01 (0.004)^*^	4.0	< 0.001	0.01	0.02	1.4% (0.7 to 2.1%)
Number of wine drinkers in household	−0.02 (0.04)	−0.5	0.650	−0.09	0.05	−1.6% (−8.2 to 5.5%)

* Significant at the *P* < 0.05 level;

** significant at the *P* < 0.01 level.

a Percentage change was estimated by exponentiating the logged estimates, then subtracting these values from 1. CI = confidence interval; SE = standard error.

Baseline consumption, guest consumption and duration of the ‘usual behaviour’ period each had a significant impact on consumption rate, as well as bottle size. Consumption rate was faster when baseline consumption was higher and if guests drank from households’ study wine and slower with each extra day spent in the ‘usual behaviour’ period (Table 4).

### Sensitivity analyses

Conclusions were unchanged with an ITT analysis which included all households that were randomized (*n* = 186), where a mixed model assumes the correlation from complete data cases should be used for the incomplete cases. (Supporting information, Data S5). The impact of bottle size on volume consumed was robust on inclusion in the analysis of the households that completed the study in reverse order to their randomization (*n* = 7), with consumption being 201.1 ml (95% CI = 51.5–349.8; *P* = 0.009) more per 14‐day period with the 75 cl compared to the 50 cl bottles.

Seventy‐three per cent (121 of 166) of households correctly guessed the study aim in response to an end‐of‐study question. The conclusions of the main analysis were unchanged by additionally including a covariate for awareness of the study aim into the analysis, which still predicted that 238.7 ml (95% CI = 71.8–404.8; *P* = 0.006) more wine was consumed per 14‐day period with the 75 cl bottles than the 50 cl bottles.

Adding a variable for participants’ self‐reported mitigating factors to the analysis, perceived to have affected their consumption during each study period, reduced the difference in consumption volume with the 75 cl bottles compared to the 50 cl bottles to a predicted 134.1 ml (95% CI = –2.8 to 269.8; *P* = 0.057).

### Content analysis of end‐of‐study questionnaire

The end‐of‐study questionnaire was returned by 169 of 173 households that completed the study. Attitudes towards the 50 cl bottles were mixed, with 44% of respondents expressing overall positive attitudes towards the 50 cl bottles, 36% overall negative attitudes and 20% being neutral or expressing positive and negative attitudes. The detailed analysis, including the main themes and subthemes that emerged from participants’ responses, is included in the Supporting information, Data S3. Statements representing the main themes are shown in Box 1.

**Box 1 Attitudes towards 50 cl bottles**

**Positive attitudes**

Right amount

*I had never thought about buying smaller volume bottles before but enjoyed drinking them as it was just the right amount to consume in an evening* (Household 111).

Drinking less

*I think I preferred the smaller bottles for regulating my drinking… I drank less with the smaller bottles* (Household 31).

*Having to open a new bottle is a mental hurdle you don't want to do and it puts you off doing so. Which I think is a positive thing… Even though the volume was the same it leads you to feel you're consuming more opening an additional bottle* (Household 86).

*It was a revelation moving over to 50 cl bottles—much nicer to have in the house and possible for two people to open and enjoy a bottle of wine without feeling the ‘pressure’ of a whole 75 cl to finish… I would feel more able to enjoy a (single) small glass of wine with my partner without having to open a huge bottle* (Household 52).

**Negative attitudes**

Value for money

*I think smaller bottles should be comparably cheaper to a big bottle… the price comparison between that and a 75 cl bottle is disproportionate* (Household 40).

*I never usually buy 50 cl bottles and think that will continue, as there is not much difference in price; therefore I think it's better value buying the bigger bottle* (Household 22).

Too small

*We tend to share one bottle of 75 cl between two people at home, the 50 cl bottle wasn't quite enough* (Household 29).

*I think I would probably drink more of the smaller bottle* (Household 111).

*It's so easy to have a large glass, and as half the bottle is gone, you think ‘I may as well finish it off’. Whereas the larger bottles, you have a large glass, and know it will take a while to finish a bottle, so you are more likely to leave it for another night* (Household 148).

*I didn't have any issues opening a second bottle, whereas with the larger bottles I would think twice* (Household 107).

## Discussion

The results of this study suggest that, among households consuming between 1.5 and 6 litres of wine a week, purchasing wine in 50 cl bottles, compared with 75 cl bottles, reduces the volume of wine consumed at home by approximately 4.5%. If sustained and replicated, the effect size of this intervention has the potential to make a meaningful contribution to reducing wine consumption at home, where the majority of wine is drunk. The uncertainty around this estimate is, however, considerable, ranging from 1.0 to 7.9%. Consumption was also approximately 5.8% slower from the smaller bottles, although again with considerable uncertainty (0.4–10.9%). Responses to the smaller bottles were mixed; liked by some for their effectiveness in reducing consumption, but disliked for their poor value for money when compared with 75 cl bottles.

The results of this study are in keeping with the robust evidence in relation to food and non‐alcoholic drinks showing that people consistently consume more from larger portions or packages [12]. There are several possible mechanisms for this ‘portion size effect’, including social and personal norms regarding how to eat or drink [25, 30]. Smaller bottles might decrease consumption by signalling a completed episode of consumption when empty, reflecting a tendency for people to consume in ‘units’ regardless of portion or package size [21]. It may also have an effect by making additional intake of wine effortful [12]. Both these mechanisms are discernible in comments from participants (Box 1).

Internationally, 75 cl is the most common size in which wine is sold, thereby setting a norm against which smaller and larger sizes are judged. By constituting two‐thirds of a 75 cl bottle, a 50 cl bottle is probably not too small to be considered a large deviation from the norm and thus provoke resistance, but small enough to reduce consumption. Wider availability of 50 cl bottles of non‐premium wines has the potential to shift this norm. Availability of wine in 50 cl bottles, however, is currently very limited. For example, less than 2% of wine stocked by the retailer used in the current study is in 50 cl bottles. 5 This means that there are very few ‘like for like’ options for people to make a switch from 75 cl bottles. In addition to increasing the availability of wine in 50 cl bottles, their placement in retail stores could also affect their likelihood of being purchased [31]. To increase their selection over 75 cl bottles, 50 cl bottles could be placed in areas associated with higher sales, including end‐of‐aisle displays and the middle of shelves [32, 33, 34].

In addition to increasing the availability of non‐premium wines in 50 cl bottles and placing them in areas of high visibility in retail stores, increasing their affordability relative to that of 75 cl bottles will be important in shifting consumption of wine towards these smaller bottles. Given the higher financial cost of producing wine in 50 cl bottles compared to 75 cl bottles (due in part to their scarcity: a barrier that would be partially mitigated were this size to become more commonly available), fiscal policies that place a higher alcohol tax on 75 cl bottles relative to 50 cl bottles would likely be needed to ensure that smaller bottles are proportionately priced in relation to 75 cl bottles. This would need to be set at absolute rates that discourage consumption, in keeping with the strong evidence that reducing the affordability of alcohol reduces its consumption [2, 35].

Although altering the placement and increasing the availability and affordability of 50 cl bottles may increase selection of this bottle size relative to 75 cl bottles, the impact of these strategies upon the total amount of wine consumed at a population level remains unknown. Among those consuming between 1.5 and 6 litres of wine a week per household, purchasing wine in 50 cl (rather than 75 cl) bottles could reduce their consumption. The impact on consumption among those consuming less than this remains unknown. While it may have a similar impact, it could increase consumption in this group if it reduced existing barriers to consumption, such as inhibitions over opening larger packages to avoid overconsumption or wastage posed by the availability of wine in larger bottles. Provided the absolute pricing of wine is set at a level that minimizes alcohol harm among populations, this concern should not be realised, but awaits empirical investigation.

The current study compared the impact of 75 cl with 50 cl bottles and provides evidence of the effectiveness of smaller bottles to reduce wine consumption. The results are potentially applicable to beer and spirits when consumed from larger compared to smaller bottles and cans. It is unknown whether bottle sizes smaller than 50 cl would decrease consumption further or have the opposite effect of increasing consumption, perhaps by encouraging consumption of multiple bottles in a drinking episode. Further research is therefore needed to assess the impact on consumption of bottles smaller than 50 cl.

The size of wine glasses used with different‐sized bottles may enhance or diminish any effect of bottle size. Larger wine glasses increase the volume of wine sold in restaurants but not in bars [36], which may reflect more free‐pouring of wine in the former, given that in restaurants more wine is typically sold by the bottle rather than by the glass. Given that wine glasses have dramatically increased in size during the last three decades [37], it seems plausible that participating households were frequently using glasses that maximized rather than minimized consumption. Drinking from smaller bottles with smaller glasses may therefore enhance the effect size observed in the current study. Future research should assess the combined effect of bottle size and glass size on wine consumption.

The main strength of the current study is that it provides the first estimate, to our knowledge, of the impact of smaller (50 cl) and larger (75 cl) bottles of wine upon consumption in the home. Further strengths include the study design (informed by a feasibility study), which included the use of standardized measures of consumption. Additionally, the high retention rate is particularly noteworthy for this type of complex intervention.

There were some limitations. First, consumption of other alcohol apart from wine was not assessed. Therefore, it is not known whether households compensated for any reduced wine consumption with the 50 cl bottles by increasing consumption of other alcoholic beverages, such as beers or spirits. Secondly, volumes of wine consumed were derived from submitted photographs, and therefore comprised an estimate of consumption. While this may have introduced some error, it is likely to have been extremely small given the rigorous method of assessment. Thirdly, the sample in the present study was predominantly white, of higher education and income and within a narrow age range. Those from minority ethnic groups, of higher deprivation and of older age were under‐represented.

Although the current results are consistent with the findings of a recent feasibility study using 75 cl and 37.5 cl bottles [26], the study requires replication and extension to address a number of unanswered questions. These include the generalizability of the current findings to other bottle sizes, populations and contexts and the potential impact on population health of increased availability and affordability of smaller bottles of wine. Crucially, further studies are needed to assess the sustainability of the effect of smaller wine bottles on consumption beyond the time‐period assessed in the current study, to assess whether effects are maintained over time.

## Conclusion

Consuming wine at home from 50 cl bottles compared with 75 cl bottles may reduce both amount consumed and rate of consumption. Increasing the availability and relative affordability of 50 cl bottles of wine has the potential to contribute to policies for reducing alcohol consumption.

## Declaration of interests

All authors have completed the Unified Competing Interest form (available on request from the corresponding author) and declare: no support from any organization for the submitted work; no financial relationships with any organizations that might have an interest in the submitted work in the previous 3 years; and no other relationships or activities that could appear to have influenced the submitted work.

## Funding

This report is independent research funded by the National Institute for Health Research Policy Research Programme (Policy Research Unit in Behaviour and Health (PR‐UN‐0409‐10109: PI: Theresa Marteau)). Additional funding was provided by Wellcome Trust for Mark Pilling's salary (a Collaborative Award in Science from Wellcome Trust (Behaviour Change by Design: 206853/Z/17/Z: PIs: Theresa Marteau, Paul Fletcher, Gareth Hollands and Marcus Munafò) and Rachel Pechey's salary (a Wellcome Research Fellowship in Society and Ethics [106679/Z/14/Z]).

## Supporting information


**Data S1** Study wine list.
**Data S2** Instructions sent to participants.
**Data S3** Content analysis of end‐of study feedback received by participants.
**Date S4** Supplementary table.
**Data S5** Supplementary analysis (Intention to treat).
**Data S6** Supplementary data plots.

## Footnotes

1 Prior to the study, all recruited households bought wine in 75 cl bottles.

2 During the running of the study, modifications were made to eligibility criteria: (1) consumption rates were capped per household at 8 × 75 cl bottles per week, due to limitations on number of 50 cl bottles which could be ordered on‐line at any one time; (2) participants were told that they could not participate if they were planning to go on holiday during any of the intervention periods (or would be spending the majority of the study period away from home); (3) due to stock issues with the smaller (50 cl) bottles, which saw discrepancies between number ordered and number available at collection, only households with a car or easy access to other means of transportation were recruited.

3 Households did not have to order additional bottles if they had finished their order.

4 At baseline, households self‐reported consuming an average of 2.6 bottles of wine, which equates to approximately 23.4 units of alcohol divided among an average of approximately two household members, i.e. around 11.7 units each.

5 At the time of checking (September 2019), 11 of more than 600 different wines were sold in 50 cl bottles.
