# Supplementary material for: Impact of bottle size on in‐home consumption of wine: a randomized controlled cross‐over trial
Source: Addiction. 2020 Apr 8;115(12):2280–92. doi: 10.1111/add.15042 (PMC8190654; doi:10.1111/add.15042)
Supplement: Supplementary file 1 — Data S1 Study wine list. Data S2 Instructions sent to participants. Data S3 Content analysis of end‐of study feedback received by participants. Date S4 Supplementary table. Data S5 Supplementary analysis (Intention to treat). Data S6 Supplementary data plots. [file ADD-115-2280-s001.docx]

**Supplementary Materials**

**S1 – Study wine list^[[1]](#footnote-2)^**

This is a simplified version of what participants saw. Participants viewed separate wine lists for 75cl and 50cl bottles and their versions included tasting notes, country of origin and links to the wine on the retailer website.

**White Wine**

**Hardys Crest Chardonnay 75Cl: £ 7.00; 50CL: £4.00**

[
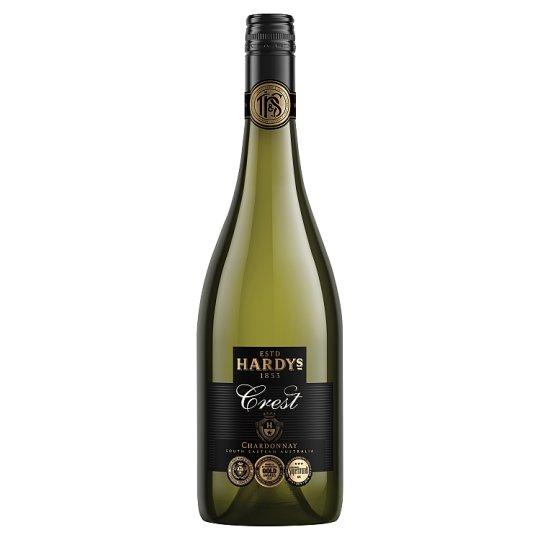
](https://www.tesco.com/groceries/en-GB/products/254819789)

**Chardonnay - White Australian Wine**

**ABV:** 13% vol

**Isla Negra Sauvignon Blanc 75Cl: £5.00; 50Cl: £3.75**

**
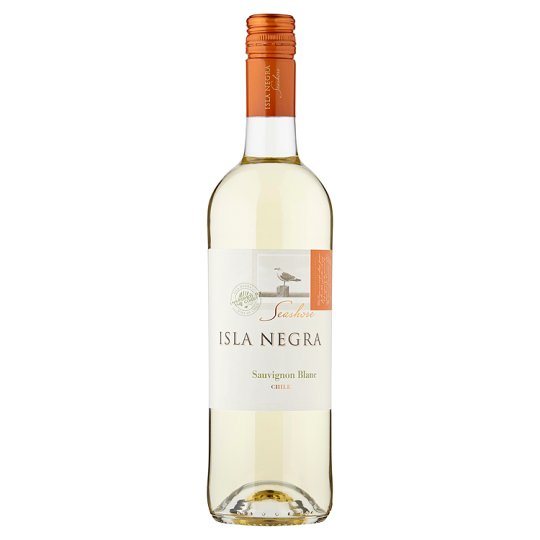
**

**Sauvignon Blanc - White Chilean Wine**

**ABV:** 12% vol

**Mud House Sauvignon Blanc 75Cl: £9.00; 50Cl: £5.00**

[
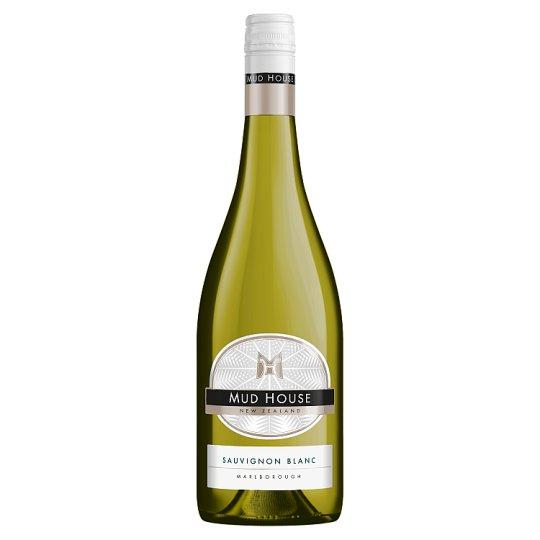
](https://www.tesco.com/groceries/en-GB/products/283840321)

**Sauvignon Blanc - White New Zealand Wine**

**ABV:** 12.5% vol

**Isla Negra Chardonnay/Px 75Cl: £5.00; 50Cl: £3.75**

[
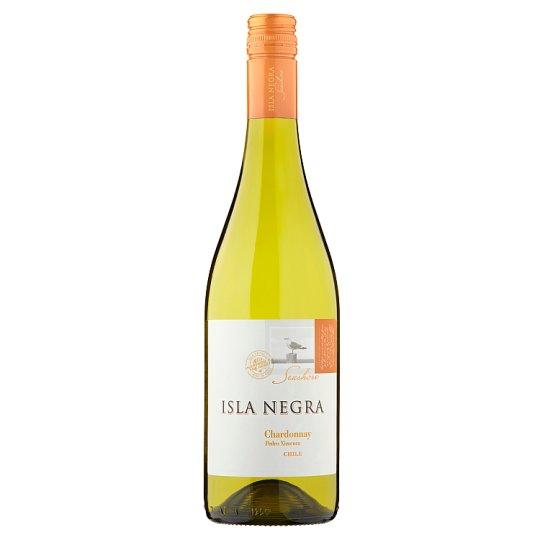
](https://www.tesco.com/groceries/en-GB/products/253805882)

**Chardonnay - White Chilean Wine**

**ABV:** 12.5% vol

**Tesco Finest Marlborough Sauvignon Blanc 75Cl: £7.50; 50Cl: £5.00**

[
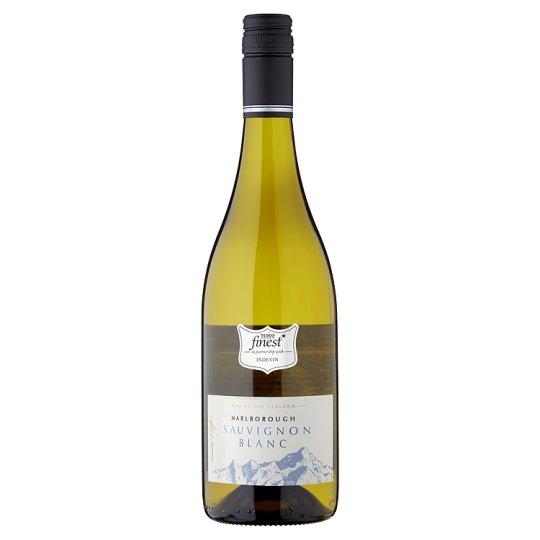
](https://www.tesco.com/groceries/en-GB/products/251108915)

**Sauvignon Blanc. Marlborough. Wine of New Zealand**

**ABV:** 12.5% vol

**Rose Wine**

**Mud House Sauvignon Blanc Rose 75Cl: £7.00; 50Cl: £5.00**


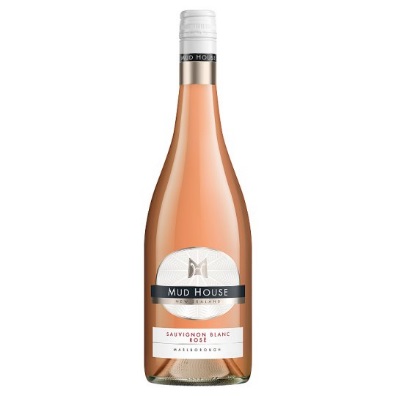


**Sauvignon Blanc - Rosé New Zealand Wine**

**ABV:** 12.5% vol

**Red Wine**

**Casillero Del Diablo Cabernet Sauvignon 75Cl: £8.00**; **50Cl: £5.00**


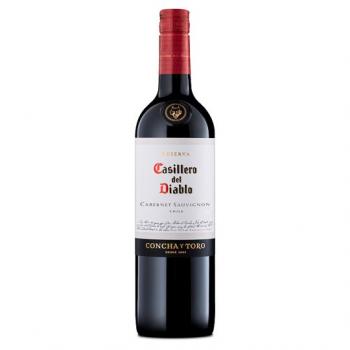


**Cabernet Sauvignon - Red Chilean Wine**

**ABV:** 13.7% vol

**Trivento Reserve Malbec 75Cl: £8.00; 50Cl: £5.00**

[
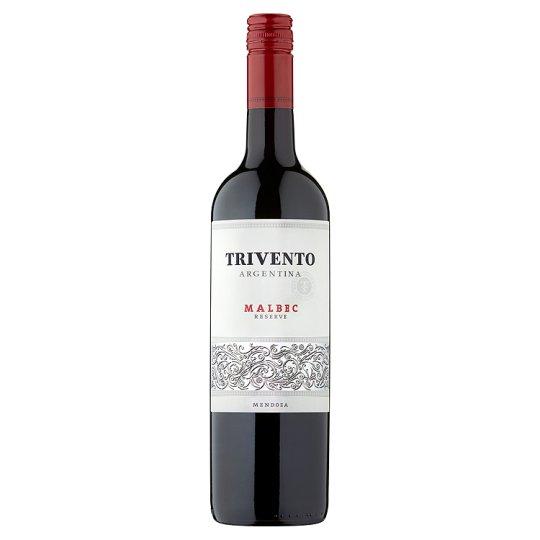
](https://www.tesco.com/groceries/en-GB/products/278363743)

**Malbec - Red Argentinian Wine**

**ABV:** 13% vol

**Tesco Finest Rioja Reserva 75Cl, £8.50; 50Cl: £6.00**

[
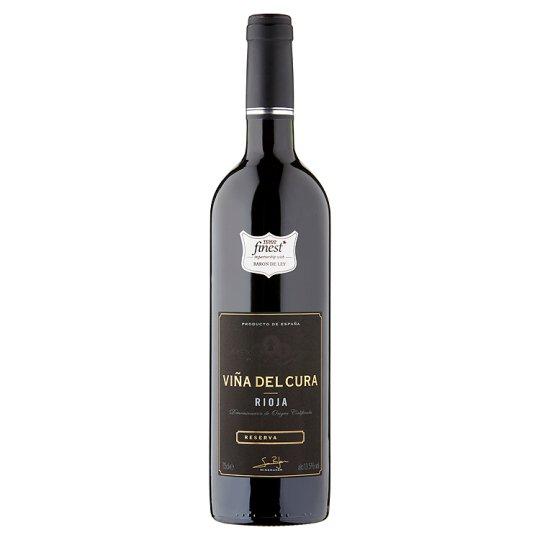
](https://www.tesco.com/groceries/en-GB/products/261787141)

**Rioja - Red wine Product of Spain**

**ABV:** 13.5% vol

**Barossa Ink Shiraz 75Cl, £10.00; 50Cl: £6.00**

[
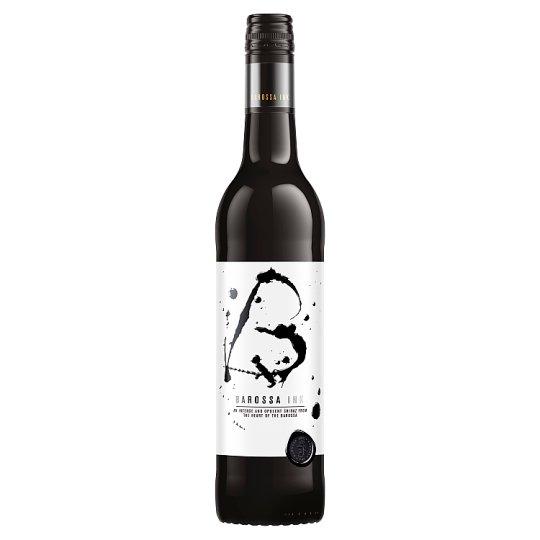
](https://www.tesco.com/groceries/en-GB/products/295459547)

**Shiraz - Red Australian Wine**

**ABV:** 14% vol

**Isla Negra Merlot Cabernet Sauvignon 75Cl: £5.00; 50Cl: £3.75**

[
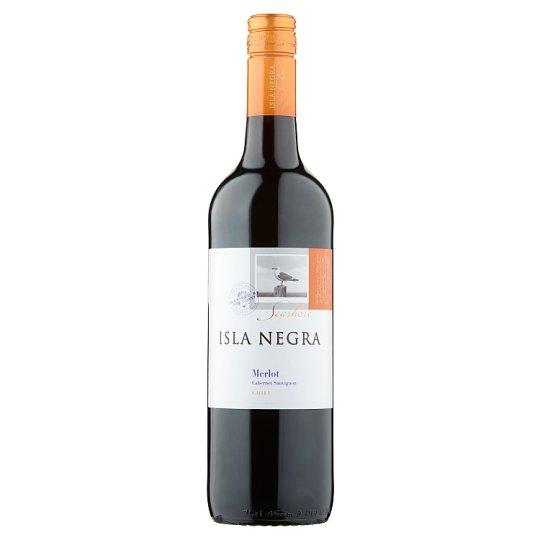
](https://www.tesco.com/groceries/en-GB/products/252954913)

**Merlot / Cabernet Sauvignon - Wine of Chile**

**ABV:** 12% vol

**Hardys Crest Cabernet Shiraz Merlot 75Cl: £7.00; 50Cl: £4.00**

[
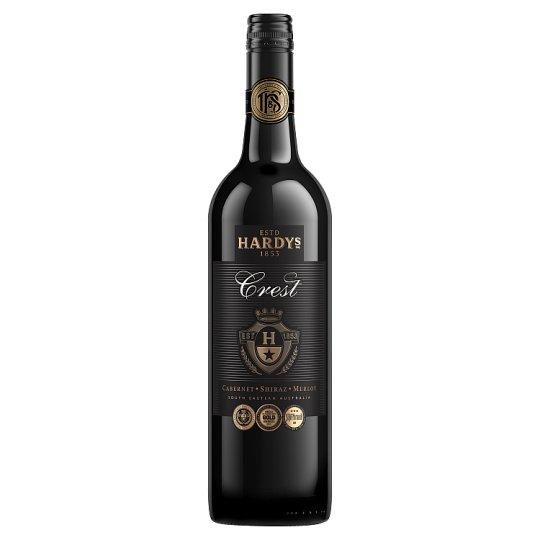
](https://www.tesco.com/groceries/en-GB/products/267787916)

**Cabernet Shiraz Merlot - Red Australian Wine**

**ABV:** 14% vol

**S2 -Instructions sent to Participants**

Date:

Participant ID: <<ID>>

Dear <<Household Representative Name>>

**Title of Study:** ‘Impact of bottle size on people’s experiences of consuming wine’

Thank you for completing the first step of the research by placing your first wine order from Tesco and sending us the order confirmation. Your account (held at Roots Research) has now been credited with £40. This sum, along with any other sums you earn during Study Period 1, will be paid by BACS/PayPal after your participation in this phase has been formally acknowledged as concluded.

Below are instructions on what you will need to do during the study. Enclosed with this letter you will find a study information sheet and some labels to stick on your wine bottles.

**We remind you that** **the study consists of three study periods.** Below you can see an illustration of the study timeline:


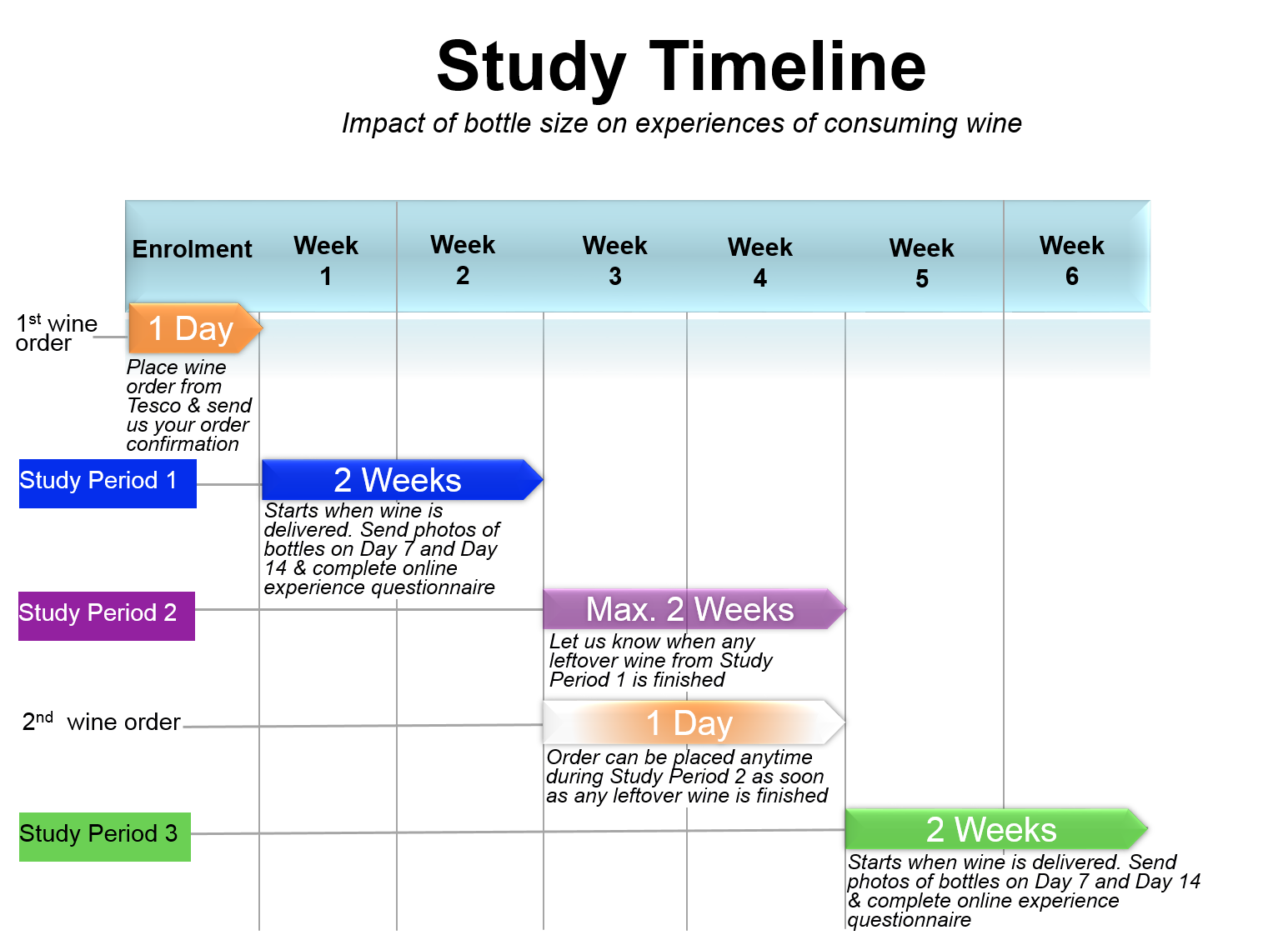


**STUDY PERIOD 1**

Receiving your wine order marks the beginning of **Study Period 1**, which will last 14 days.

*Please note: to help you with preparing, affixing, and completing the labels we have created a short video. We recommend that you take the time to view this video as the instructions contained will help to ensure that your photos can be accepted on first submission. Link to video:* [*https://www.youtube.com/watch?v=_zSIWJsciXw&feature=youtu.be*](https://www.youtube.com/watch?v=_zSIWJsciXw&feature=youtu.be) *(also has been sent in confirmation of order email).*

**During this time, you will need to:**

- Stick the enclosed labels on your wine bottles (one label per bottle). Instructions on how to affix the labels on the bottles are on page 4
- Use the labels to record:

1. the date and time each bottle was opened and finished
2. the number of people, including any guests, who drank from each bottle (Instructions on how to fill-in the labels are on page 5)

- Drink the study wine as you normally would
- **NOT** buy or drink any other wine while at home for the duration of Study Period 1
- **Retain your empty wine bottles until you have photographed them** and sent us your photographs
- Send us close-up photographs of each bottle of study wine (regardless whether bottles are empty, partially empty, or full) on two occasions: i.e. 7 days and 14 days after you receive your wine order. Instructions on how to take these photos are detailed on pages 6-8
- Empty bottles can be discarded after they have been photographed at 7 days. No need to photograph them again at 14 days
- Complete an online questionnaire on your experience of drinking the wine 7 days and 14 days after you receive your wine order. We will email you the link to these questionnaires

**You need to send us your photographs on day 14 to be able to continue with the next study periods and receive full compensation.**

**Please let us know during this period if** you are running low on wine and need to place an additional order to last you for Study Period 1.

**STUDY PERIOD 2**

**Study Period 2** is a two-week gap between periods 1 and 3, where we give you time to finish any remaining wine from your first wine order, before placing your second wine order. You should place and receive your second wine order within 21 days of finishing Study Period 1.

**During this time, you will need to:**

- Notify us by email as soon as your wine is finished or close to finishing (i.e. you have started your last bottle). Ideally you should receive your second wine order as soon as the wine from your first order is finished, so that you are not left without any wine
- Visit our study website to choose your wines, just as you did for Study Period 1. We will send you the link to the study website once we receive your notification. By entering your participant ID into this site, you will be informed of how many bottles you will need to order, and in which size
- Send us confirmation of your second wine order

**Your second wine order should be placed and your wine received within 21 days (3 weeks) of finishing Study Period 1.**

**If you have not received your second wine order in this timeframe, your participation will be ended and you will receive no further compensation.**

If we have not heard from you within two weeks of the end of Study Period 1, we will contact you to ask you about any leftover wine you have.

**STUDY PERIOD 3**

Receiving your second wine order will mark the beginning of **Study Period 3**, which will last 14 days. **During this time, you will need to repeat the procedures you followed during Study Period 1:**

- Stick the enclosed labels on your wine bottles (one label per bottle). Instructions on how to affix the labels on the bottles are on page 4
- Use the labels to record:

1. the date and time each bottle was opened and finished
2. the number of people, including any guests, who drank from each bottle (Instructions on how to complete the labels are on page 5)

- Drink the study wine as you normally would
- **NOT** buy or drink any other wine while at home
- **Retain your empty wine bottles until you have photographed them** and sent us your photographs
- Send us close-up photographs of each bottle of study wine (empty, partially full and unopened) on two occasions: 7 days and 14 days after you receive your wine order. Instructions on how to take these photos are detailed on pages 6-8
- Empty bottles can be discarded after they have been photographed at 7 days. No need to photograph them again at 14 days
- Complete an online questionnaire on your experience of drinking the wine 7 days and 14 days after you receive your wine order. We will email you the link to these questionnaires.

**You need to send us your photographs on day 14 to be able to receive full compensation.**

During Study Periods 1 and 3, we will send you reminders via emails asking you to send us your photographs on the morning of the day they are due. If we don’t hear from you, we will send you follow-up reminders.

**Compensation**

You will receive a **maximum of £240**, **if you complete all aspects of the study**. Below is a breakdown of how much you will receive for each task you complete. You will receive payment from Roots Research via PayPal or bank transfer on two occasions: within two weeks of completing Study Period 1 and within two weeks of completing the study.

**STUDY PERIOD 1**

- *Ordering wine and providing confirmation of order:* £40

(This amount will also cover delivery charges and minimum order costs)

- *Sending photographs of all bottles of ordered wine on:*
- Day 7: £30
- Day 14: £50

**STUDY PERIOD 2**

*Ordering wine and providing confirmation of order:* £50

(This amount will also cover delivery charges and minimum order costs)

**STUDY PERIOD 3**

*Sending photographs of all bottles of ordered wine on:*

- Day 7: £30
- Day 14: £40

You might also be selected to complete an of end-of-study telephone interview, in which case you will receive an additional £20

**How to affix study label on each bottle**

1. **Affix a label on each bottle you receive**
2. Ensure that the **left edge of the label is aligned with the left edge of the pre-existing manufacture’s label** (see photo below)
3. **Give each label a number in the number box,** e.g.: if you order 10 bottles in your first order, then give each bottle a number from 1 to 10 (this does not have to relate to the order you drank them in).

**
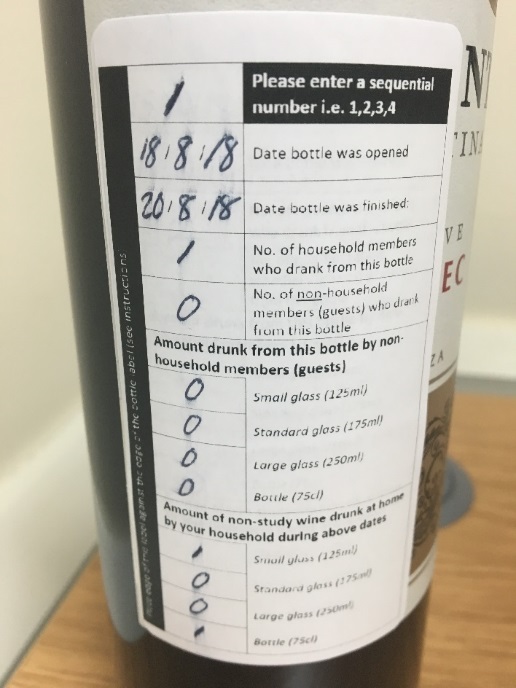
**

Example of how to correctly affix and complete the study label

**How to complete the study labels**

1. Use a ballpoint pen to fill in the labels to avoid your answers rubbing off or smudging.
2. Try to keep a note of any wine that you give to anyone not part of your household – *i.e.* guests - and any non-study wine you and your household members drink while at home.
3. Try to stick the study label on the side of the bottle, partly overlapping with the manufacturer label

**Complete each label it as follows:**

(see photograph on previous page for example of completed label)

1. Add a sequential number corresponding to number of bottles in your delivery

**The label**

|  |  | **Please enter a sequential number i.e. 1,2,3,4** |
| --- | --- | --- |
|  | / / | Date bottle was opened |
|  | / / | Date bottle was finished: |
|  |  | No. of household members who drank from this bottle |
|  |  | No. of non-household members (guests) who drank from this bottle |
|  | **Amount drunk from this bottle by non-household members (guests)** | |
|  |  | *Small glass (125ml)* |
|  |  | *Standard glass (175ml)* |
|  |  | *Large glass (250ml)* |
|  |  | *Bottle (75cl)* |
|  | **Amount of non-study wine drunk at home by your household during above dates** | |
|  |  | *Small glass (125ml)* |
|  |  | *Standard glass (175ml)* |
|  |  | *Large glass (250ml)* |
|  |  | *Bottle (75cl)* |

1. How many people from your household, including yourself, drank from this bottle?
2. Fill in the date you opened this bottle and the date you finished it.
3. How many people not part of your household (i.e. guests), drank from this bottle?
4. Add the serving size for any wine drunk by guests, e.g.:

Small glass (125ml): 1

Standard glass (175ml): 2

Large glass (250ml): 0

If your guests drank the whole bottle, add a “YES” next to the ‘Bottle (75cl)’ option.

1. If any of your household members, including yourself, drank any non-study wine while at home, between opening and finishing this bottle, add the number drunk next to each option. For example:

Small glass (125ml): 0

Standard glass (175ml): 2

Large glass (250ml): 0

Bottle (75cl) :1

**How to take the photographs:**

On days 7 and 14 of Study Periods 1 and 3 you will need to send us photographs of your study wine.

During each of these times you need to:

1. **Take a photo of each individual bottle (all bottles: empty, partially full and unopened) separately**. Do not take one photo of all bottles together. If, for example, you were asked to order 12 bottles of wine, you will need to send us at least 12 photographs on each occasion.
2. Ensure each photo is taken **at least 30cm or more away from the bottle**
3. Take photos against a **white, or pale background**
4. **Ensure that** **the entire bottle is clearly visible** – including its full base and whole lid
5. The photos should be of the **side view of the bottle** i.e. **NOT showing all of any manufacturer labels** on the bottle
6. Make sure the **study** **label** you affix on each bottle **is clearly visible and readable** or that we can zoom in and read it. If you take a photograph of the entire bottle and feel the label is not clearly visible, take a close-up photo of the label as well.
7. Make sure the photos are **clear** and **NOT** **blurry**

On the next page follows examples of acceptable and unacceptable photos.

**Examples of ACCEPTABLE photos**

| **Large wine bottle example** | **Small wine bottle example** |
| --- | --- |
| **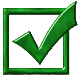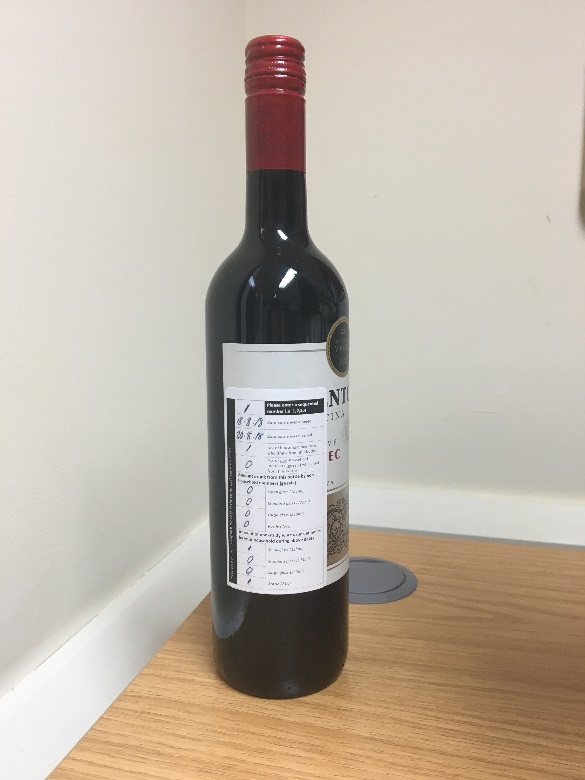** | **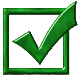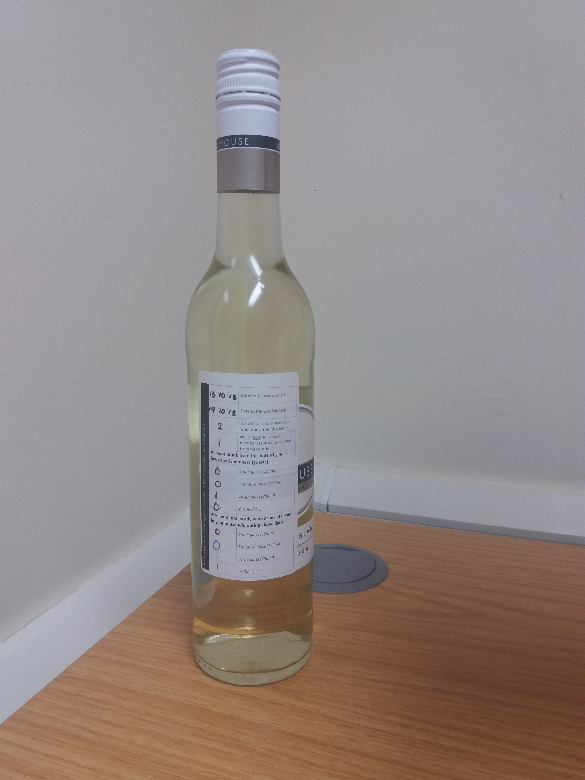** |

*Photos are not blurry and the study label is clearly visible, readable and allows us to zoom in and view it more closely. One bottle is presented per image. The entire bottle is visible. The study label is not placed entirely over the front of the manufacturer’s label, allowing the sides of the bottle to be visible*

**Examples of UNACCEPTABLE photos:**

| **Problem:** Image is blurry | **Problem:** Close up of label only– entire bottle not visible |
| --- | --- |
| **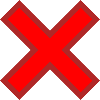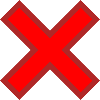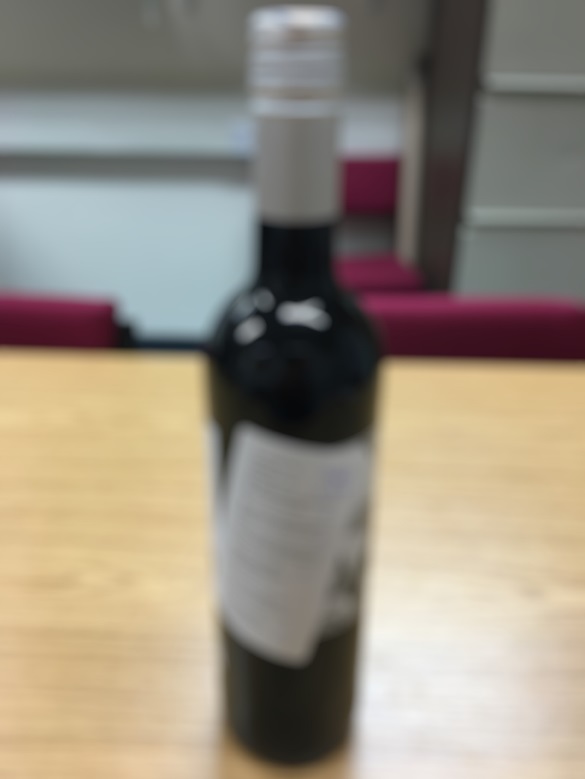** | **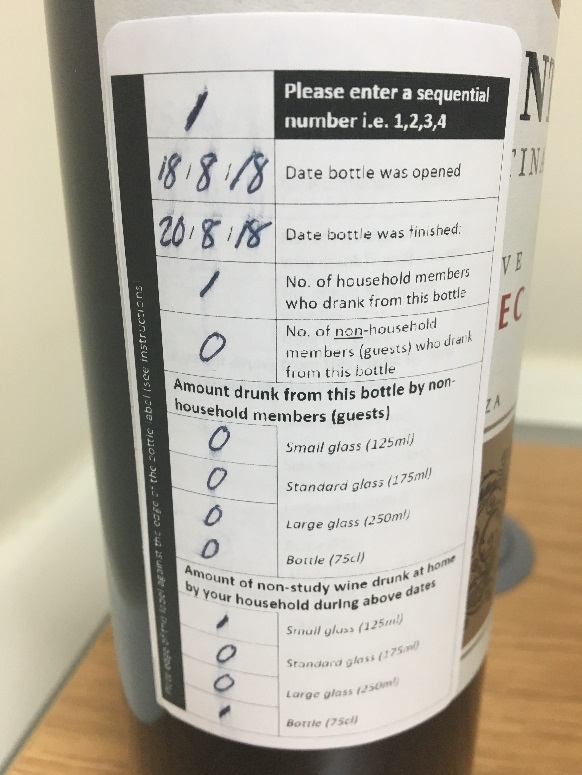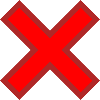** |

| **Problem:** Side view of the bottle not visible | **Problem**: Many bottles in image – the bottles behind are not visible |
| --- | --- |
| 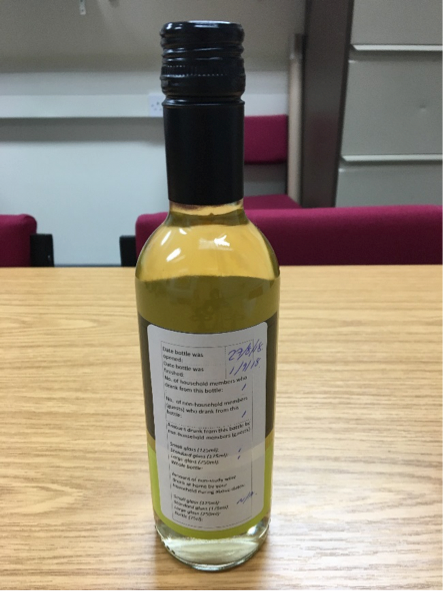 | **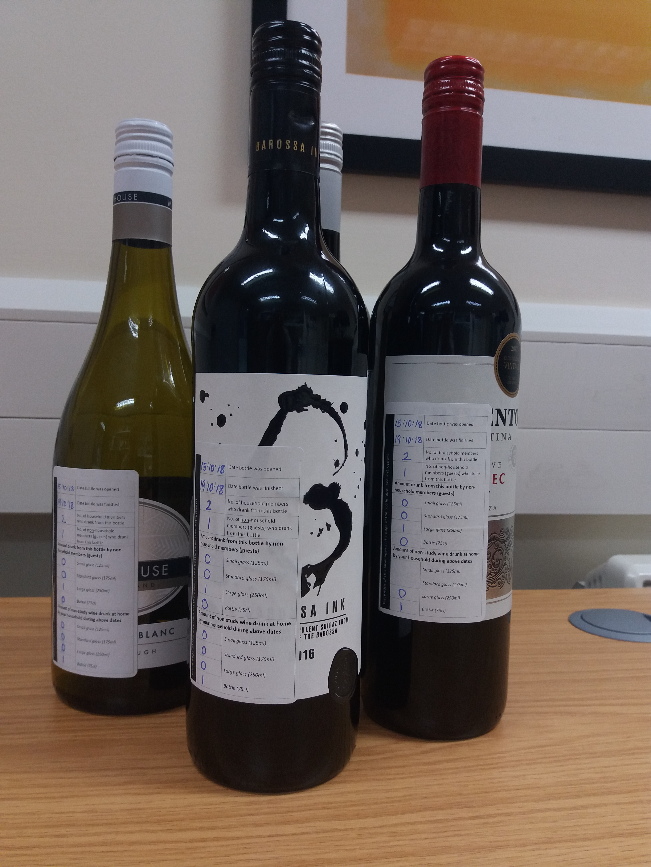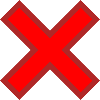** |
| **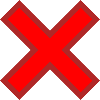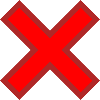**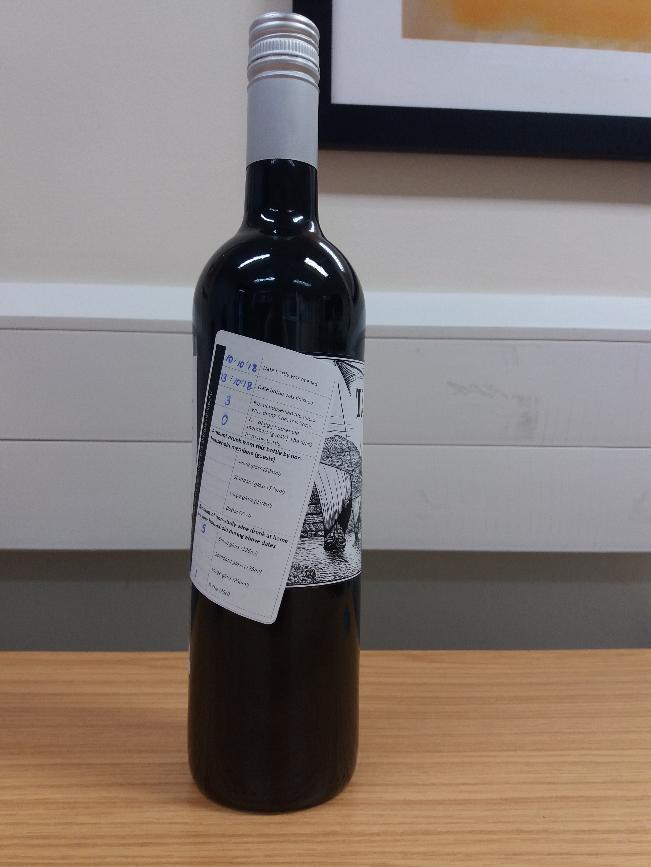 | + |
| **Problem**: Label has been placed at an angle obstructing full side view of bottle |  |

If you have any questions about these instructions, or are unclear about anything else in the study, please contact the research team via email: (email removed) or on (number removed)**.**

**How to send photos to our email address or mobile number**

You can send photos taken on your phone to the study team via email, WhatsApp (free on Wi-Fi) or text message. You may incur charges when sending photos via text message, so please think about your preferred method. If you’re not sure what will work best for you, please feel free to try any method.

**Send emails with pictures to:** (email removed)

- If you want to email the image to us, put your Participant ID in the Subject Line (found at the top of this letter), so that we can check that the images we receive are yours.

**Send WhatsApp message with pictures to:** **(number removed) WB Research**

- To do this, ensure you have WhatsApp installed on your phone and then save our number to your contacts / address book on your phone (using **WB Research** as the contact name). Please add your Participant ID to the message (found at the top of this letter).

**Send text message with pictures to: (number removed) WB Research**

- If you want to text the image to us, before hitting ‘send’ please add your Participant ID to the message (found at the top of this letter) so that we can check that the images we receive are yours.

**Further instructions for how to send a photo taken on your mobile phone**

1. Depending on the type of phone you have, after taking a photo you may return to your camera and your gallery will have the new image. If this happens, please select the picture you just took (it may be at the top or bottom of your screen) in order to go to the gallery.
2. When you’re in your gallery, you may need to click the image you want to send to us again, in order to see the ‘share’ symbol (which could look like this
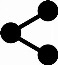

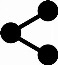

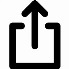
 [
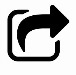
](https://www.bing.com/images/search?view=detailV2&ccid=FgshjIvg&id=A481169811534ED054081C9385E4453BBE752814&thid=OIP.FgshjIvgPwYzzEoj1DU24AHaHa&mediaurl=http://www.free-icons-download.net/images/share-share-icon-63786.png&exph=512&expw=512&q=share+icon&simid=608053945616043972&selectedIndex=24)) or an ‘options’ symbol (which could look like this
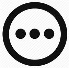
 ) at the top or bottom of your screen.
3. If you see the ‘share’ icon, click on it and then select the way you want to send the image to us – i.e. via email, text message or another messaging service.
4. If you see the ‘options’ icon, click on it and then select ‘Share via…’. You will then be able to select the way you want to send the image to us – i.e. via email, text message or another messaging service
5. Your text message or email system will need to have access to your photos in order to directly send the image – so don’t worry if your phone asks for permission to do this.
6. When you have selected the way you want to send the image to us – the selected programme will open.
   - If you want to email the image to us, enter (email removed) in the ‘To’ line and put your Participant ID in the Subject Line.
   - If you want to text, or WhatsApp the image to us, enter our mobile number (removed). For WhatsApp you will need to add the number to your contacts first (see instructions above). Before hitting ‘send’ **please add your Participant ID to the message.**

If you have any questions about sending photos, please contact the research team on **(number removed)**, or via email (email removed).

**S3 – Content analysis of end-of study feedback received by participants**

**Perceptions of 50cl wine bottles**

Participants were requested to complete an end-of study questionnaire enquiring about bottle size preferences, whether their consumption increased with either of the two bottle sizes and what they thought the study was about. Responses were analysed with a focus on perceptions towards the 50cl bottles. These responses were coded in terms of their overall tone towards the bottles as positive, negative or neutral/mixed. Main themes and subthemes across comments were also identified and classified as positive, negative or neutral/mixed.

Results

169 households completed or partially completed the end-of-study questionnaire, including 162/166 of the households that completed the study and were included in the main analysis and 7 that completed the study but in the reverse order to their randomisation.

44% of the comments were categorised as positive towards the 50cl bottles; 36% as negative and 20% as neutral or mixed.

**Positive**

Main positive themes that emerged were:

1. **General positivity**

Participants often described the 50cl bottles as ‘nice’, ‘cute’, ‘aesthetically pleasing’ and ‘generally a good idea’ (“*The small bottles look more attractive, a bit like small shoes”* (Household 28)).

1. **Convenience**

The most commonly cited convenience of using the 50cl bottles was related to their i) ***amount,*** which was often described as ideal *(“I had never thought about buying smaller volume bottles before but enjoyed drinking them as it was just the right amount to consume in an evening”* (Household 111)).

Other issues discussed in relation to the convenience of using 50cl bottles were: ii) ***ease of pouring wine*** *(“not as heavy whilst pouring”* (Household 49))*,* iii) ***easier*** ***storage*** (“*50cl, easier to fit in the fridge!”* (Household 107)); iv) ***ideal for cooking*** *(“I like the small bottles and are a good size if cooking etc”* (Household 26)) and v) ***less wine wastage*** (i.e. due to remaining wine in bottle going bad) (“*Having this smaller bottle means we can enjoy fresh wine when it’s at its best just after opening, without wasting it or having to leave until the next day when it won’t taste as good”* (Household 66)).

1. **Decreasing wine consumption**

The 50cl bottles were often perceived as decreasing wine consumption (“*I think I preferred the smaller bottles for regulating my drinking…. I drank less with the smaller bottles*” (Household 31)).

As such they were sometimes described as ***good for*** ***health*** *(“I would prefer to buy the 50cl …Better calories wise as well if you are on a diet.”* (Household 72); *“…for Health reasons I prefer the smaller bottle” (*Household 86)).

1. **Factors facilitating reduced consumption**

The main mechanisms emerging from participants’ accounts by which 50cl bottles were perceived as facilitating reduced wine consumption were:

1. ***Increasing control and awareness of consumption***

The 50cl bottles were perceived as allowing individuals to control their drinking (*“I could control my drinking habits better”* (Household 58)), often through the increased number of empty 50cl compared to 75cl bottles, which increased awareness of consumption levels *(“as the empty 50cl bottles mounted up pretty quickly compared to the 75cl. It looks awful if you drink regularly”* (Household 85)).

1. ***Inhibiting opening of multiple bottles***

Participants described being less likely to open an additional bottle once a 50cl bottle was finished *(“I preferred the smaller size bottles - made you think twice before opening another bottle and having another glass of wine”* (Household 51); ”*I usually have no problem drinking 3 glasses in an evening however there were exactly 2 glasses in the small bottle. When I went for the 3rd glass and had to open another bottle I felt almost guilty for opening another bottle...I knew it was a smaller bottle but the idea of opening another bottle made me think I had drunk too much. With a big bottle I don't really notice how many glasses I drink, with the smaller bottle I knew exactly how much I had drunk”* (Household 79)).

Opening a new bottle was described as a ‘mental hurdle’ *(“Having to open a new bottle is a mental hurdle you don’t want to do and it puts you off doing so. Which I think is a positive thing…. Even though the volume was the same it leads you to feel you’re consuming more opening an additional bottle”* (Household 86)).

1. ***Less amount available per bottle***

Consumption was often described as being by the bottle regardless of size *(“As its quite normal to just finish a bottle of wine. Whereas it takes more conscious effort to open another bottle. You can just set your limit as a bottle of wine, rather than setting it in terms of cl”* (Household 138)). This meant that less wine was consumed with 50cl bottles compared to 75cl bottles *(“I didn't want to open another bottle - …2 large glasses were enough whereas if I opened a 70cl bottle I would probably drink the whole thing” (*Household 81); “*There's a tendency if the bottle is open that it should be finished, and with a 50cl bottle there's less to get through” (*Household 139)).

On the contrary, participants described finishing the amount of wine in a 75cl bottles because it was available (“*I think the larger 75ml bottle size encourages you to drink more as once the bottle is opened you feel you should drink it so you do not waste the wine…”* (Household 61); “*I'm not totally sure why but potentially because the bottle (75cl) has more wine so I felt it OK to drink more and there was more readily available without needing to open up a new bottle..”* (Household 18)).

This was related to the amount in the 50cl bottles, which was often considered adequate. (“*it was a revelation moving over to 50cl bottles - much nicer to have in the house and possible for 2 people to open and enjoy a bottle of wine without feeling the 'pressure' of a whole 75cl to finish…50cl as I would feel more able to enjoy a (single) small glass of wine with my partner without having to open a huge bottle”* (Household 52); “*The 70cl bottles encouraged me to drink more as I would want to finish the bottle, having a smaller bottle I only drank what I needed to*” (Household 103)).

**Negative**

Main negative themes emerging were:

1. **Convenience**
2. ***Value for money***

The most dominant negative theme emerging in relation to the 50cl bottles, even from responses with an overall positive tone, was their cost and how it was disproportionate to the price of a 75cl bottle (“*I think smaller bottles should be comparably cheaper to a big bottle. … the price comparison between that and a 75cl bottle is disproportionate.”* (Household 40)). The cost was described as putting people off buying the 50cl bottles in the future (“*the larger may be better value for money …. the price needs to be right or I'd think I'll just get a bigger one”* (Household 38); “*I never usually buy 50cl bottles and think that will continue as there is not much difference in price therefore I think it's better value buying the bigger bottle”* (Household 22)).

1. ***Insufficient amount***

Although many participants described the amount in the 50cl bottles as ideal, there were others that described dissatisfaction and thought the bottles were too small (“*500ml not so great as you only get one and bit glasses each out of it which me and my partner didn’t prefer that as we usually have two glasses of wine each out of one bottle”* (Household 1)). This was especially true for participants who tended to share a bottle rather than drink it themselves (“*We tend to share one bottle of 75cl between 2 people at home, the 50cl bottle wasn’t quite enough*” (Household 29)).

1. ***Packaging/waste***

Some participants also described how the 75cl bottles were more convenient than the 50cl bottles, predominantly due to the former requiring less packaging for the same volume of wine, resulting in less waste and thus being more environmentally friendly *(“plus easier (75cl) to carry and maybe more environmentally friendly”* (Household 144)).

1. **Increasing consumption**

Many participants perceived the 50cl bottles to have increased their wine consumption (“*I think I would probably drink more of the smaller bottles”* (Household 111)).

1. **Factors facilitating consumption**

The main mechanisms emerging from participants’ accounts by which 50cl bottles were perceived as facilitating wine consumption were:

1. ***Finishing off bottle/not worth keeping leftovers***

Participants described how they were more likely to finish off a 50cl bottle in one sitting rather than save any leftover wine for another time (“*it’s so easy to have a large glass, and as half the bottle is gone, you think "I may as well finish it off". Whereas the larger bottles, you have a large glass, and know it will take a while to finish a bottle, so you are more likely to leave it for another night”* (Household 148)).

1. ***Opening second bottle***

In addition to being more likely to finish a 50cl bottle, many participants described being inclined to open a second or multiple 50cl bottles in one evening, something they wouldn’t do with the 75cl bottles (“*I didn't have any issues opening a second bottle whereas with the larger bottles I would think twice*” (Household 107))*.* Opening and finishing a second 50cl bottles resulted in more wine being consumed overall with these bottles (i.e. 100cl vs 75cl out of the large bottles) *(“not so guilty at opening another one as it was not a full bottle, but defeated the object if we got through 2 smaller bottles instead of 1 normal bottle”* (Household 165)).

1. ***Increasing drinking occasions***

The 50cl bottles were sometimes perceived as increasing the drinking occasions. Some participants described how 50cl bottles allowed them to consume wine in situations that they otherwise wouldn’t (e.g. mid-week, lunchtime), as a 75cl seemed too big to open *(“50cl - we were more likely to open it and have a drink during the week rather than only Fridays and at the weekend - however not so good for having no days of drinking as is recommended”* (Household 153)).

1. ***Perceived amount***

Finishing a 50cl bottle in one sitting and opening a bottle when one would otherwise not, were related to the perceived amount of wine in the bottles. The 50cl bottles were sometimes perceived as smaller than they were *(“I was drawn towards drinking the whole 50cl bottle. They seemed smaller than they were”* (Household 57); “*finishing a bottle felt less impactful (or a big deal) with small bottles, whereas in a larger bottle this almost signified an 'end of the night', whereas in the smaller ones we felt as if we should keep drinking*” (Household 3)) or caused dissatisfaction due to insufficient amounts *(“I didn't feel satisfied with one bottle”* (Household 82)).

**Neutral/Mixed**

The main mixed/neutral themes that emerged were:

1. **Amount**

Whether or not the amount in a 50cl bottle was considered sufficient was often described as depending on the situation or occasion *(“I would definitely consider buying the smaller bottles for some evenings, for example if we just wanted a glass with dinner. … I’d buy both, sometimes I’d prefer the larger bottle and other times the smaller bottle, depending on my plans”* (Household 150)). Generally, the 50cl bottles were considered ideal for one or two people sharing, and the 75cl bottles for larger groups *(“I preferred the 50cl for drinking by myself or in the weekdays. However, when I had guests I preferred the 75cl bottles”* (Household 138)). In consequence, whether or not the 50cl bottles were considered as having had an impact on consumption was sometimes described as depending on the context, environment or situation *(“I think I drank more when by myself, (with 50cl bottles) i.e. a glass a day with dinner. But when with my girlfriend I drank less, as opening 2 bottles of 50cl is too much wine but 1 bottle of 50cl isn't enough” (*Household 24)).

1. **Impact on consumption**

Some participants reported that bottle size did not have an impact on their wine consumption *(“I believe that I consumed the same amount from both bottles”* (Household 117)).

**S4 – Supplementary Table**

**Table 1:** Characteristics of (a) households and (b) household representatives discontinuing participation (n=13)

| 1. **Households** | |
| --- | --- |
| **No of adults (mean (sd))** | 2.1 (1.1) |
| **Age (household) (mean (sd))** | 32.8 (6.3) |
| **No of wine drinkers (mean (sd))** | 2.1 (1.1) |
| **No of bottles consumed a week (mean (sd))** | 3.4 (1.3) |
| **Annual household income** **(n (%))** |  |
| under £15k | 0 |
| £15-£25k | 1 (8%) |
| £25-£35 | 3 (23%) |
| £35-£50k | 4 (31%) |
| £50-£70k | 1 (8%) |
| Above £70k | 4 (31%) |
| Prefer not to say | 0 |
| (**b) Household representatives** | |
| **Age (participant) (mean (sd))** | 32.4 (8.2) |
| **Sex (n (%))**  Female  Male | 5 (38%)  8 (62%) |
| **Education (n (%))**  Below A levels  A levels and vocational training  Degree and above | 1 (8%)  6 (46%)  6 (46%) |
| **Ethnicity** **(n (%))**  White  Black  Asian  Mixed | 10 (77%)  0  1 (8%)  2 (15%) |

**S5 – Supplementary analysis (Intention to treat)**

**Table 2:** Mixed-effect regression model estimates (95% CI) for volume (ml) of wine consumed per 14-day period (ITT n=186)

|  | **Estimate (SE)** |  |  | **95% CI for estimate** | | | |
| --- | --- | --- | --- | --- | --- | --- | --- |
|  |  | **t value** | **p value** |  | **Lower** | | **Upper** |
| **Intercept** | 207.9 (541.0) | 0.38 | 0.701 | -839.9 | | 1256.5 | |
| **Bottle size 75cl**  **(ref: 50cl)** | 201.1 (76.7)* | 2.62 | 0.009 | 51.5 | | 349.8 | |
| **Intervention period**  **(ref: period 1)** | -61.4 (74.3) | -0.83 | 0.410 | -206.5 | | 82.6 | |
| **Intervention order**  **(ref: 50cl first)** | 15.9 (192.4) | 0.08 | 0.934 | -356.7 | | 388.2 | |
| **Baseline consumption (ml)** | 2.21 (0.16)** | 14.0 | <0.001 | 1.91 | | 2.52 | |
| **Guest consumption (ml)** | 0.56 (0.10)** | 5.79 | <0.001 | 0.37 | | 0.75 | |
| **Out-of-home consumption (ml)** | -0.05 (0.06) | -0.844 | 0.400 | -0.17 | | 0.07 | |
| **Non-study wine consumption at home (ml)** | 0.006 (0.076) | 0.07 | 0.941 | -0.14 | | 0.15 | |
| **Price (£) per litre (L)** | 2.14 (39.7) | 0.05 | 0.957 | -75.9 | | 79.1 | |
| **‘Usual behaviour’ period duration (days)** | -48.2 (14.1)** | -3.41 | <0.001 | -75.6 | | -20.8 | |
| **Number of wine drinkers in household** | -23.6 (148.3) | -0.16 | 0.874 | -310.4 | | 263.5 | |
|  |  |  |  |  |  |  |  |

* Significant at the p<0.05 level

** Significant at the p<0.01 level

**S6- Supplementary data plots**

**Plot 1:** Adjusted mean consumption (ml) according to bottle size (95% CI)


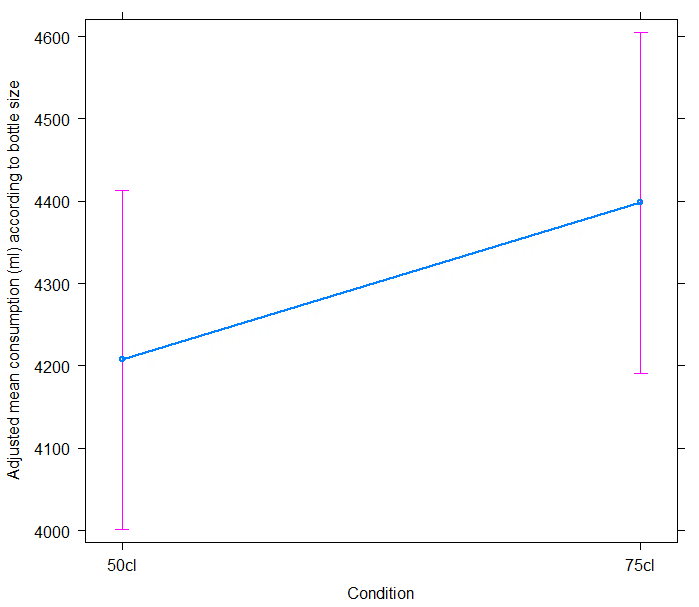


**Plot 2:** Unadjusted mean consumption (ml) for each bottle size according to intervention order (+/-1SE)


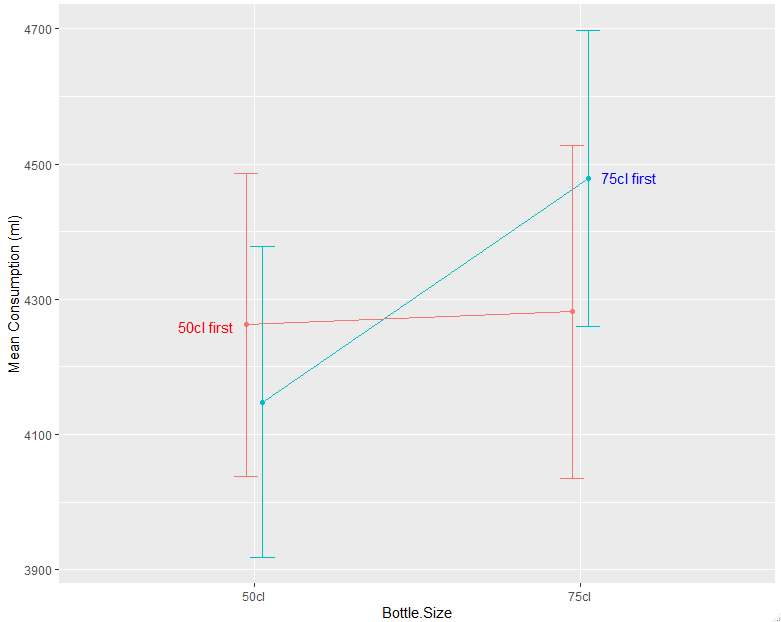


Raw data suggest that the effect of wine bottle size on the primary outcome may be greater in households receiving 75cl bottles first (Plot 2), although order did not contribute significantly to adjusted estimates in our model (t(-1.08; p=0.278; Table 3 main manuscript). If an order effect were replicated in a study suitably powered to detect it, this would suggest that reducing wine bottle size from 75cl to 50cl has a greater effect than vice versa. Alternatively, it would suggest that the wine is more salient to participants during the first intervention period, which may lead them to consume more. A future trial might want to use a settling-in period to rule this possibility out.

1. **All wines were available in both 75cl and 50cl bottles** [↑](#footnote-ref-2)
